# External Validation and Updating of a Statistical Civilian-Based Suicide Risk Model in US Naval Primary Care

**DOI:** 10.1001/jamanetworkopen.2023.42750

**Published:** 2023-11-08

**Authors:** Michael A. Ripperger, Jhansi Kolli, Drew Wilimitis, Katelyn Robinson, Carrie Reale, Laurie L. Novak, Craig A. Cunningham, Lalon M. Kasuske, Shawna G. Grover, Jessica D. Ribeiro, Colin G. Walsh

**Affiliations:** 1 Department of Biomedical Informatics, Vanderbilt University Medical Center, Nashville, Tennessee; 2 Department of Anesthesiology, Vanderbilt University Medical Center, Nashville, Tennessee; 3 Retired, US Navy, Portsmouth, Virginia; 4 Daniel K. Inouye Graduate School of Nursing, Uniformed Services University of the Health Sciences, Bethesda, Maryland; 5 Naval Medical Center Portsmouth, US Navy, Portsmouth, Virginia; 6 Department of Psychology, Florida State University, Tallahassee; 7 Department of Medicine, Vanderbilt University Medical Center, Nashville, Tennessee; 8 Department of Psychiatry, Vanderbilt University Medical Center, Nashville, Tennessee

## Abstract

**Importance:**

Suicide remains an ongoing concern in the US military. Statistical models have not been broadly disseminated for US Navy service members.

**Objective:**

To externally validate and update a statistical suicide risk model initially developed in a civilian setting with an emphasis on primary care.

**Design, Setting, and Participants:**

This retrospective cohort study used data collected from 2007 through 2017 among active-duty US Navy service members. The external civilian model was applied to every visit at Naval Medical Center Portsmouth (NMCP), its NMCP Naval Branch Health Clinics (NBHCs), and TRICARE Prime Clinics (TPCs) that fall within the NMCP area. The model was retrained and recalibrated using visits to NBHCs and TPCs and updated using Department of Defense (DoD)–specific billing codes and demographic characteristics, including expanded race and ethnicity categories. Domain and temporal analyses were performed with bootstrap validation. Data analysis was performed from September 2020 to December 2022.

**Exposure:**

Visit to US NMCP.

**Main Outcomes and Measures:**

Recorded suicidal behavior on the day of or within 30 days of a visit. Performance was assessed using area under the receiver operating curve (AUROC), area under the precision recall curve (AUPRC), Brier score, and Spiegelhalter *z*-test statistic.

**Results:**

Of the 260 583 service members, 6529 (2.5%) had a recorded suicidal behavior, 206 412 (79.2%) were male; 104 835 (40.2%) were aged 20 to 24 years; and 9458 (3.6%) were Asian, 56 715 (21.8%) were Black or African American, and 158 277 (60.7%) were White. Applying the civilian-trained model resulted in an AUROC of 0.77 (95% CI, 0.74-0.79) and an AUPRC of 0.004 (95% CI, 0.003-0.005) at NBHCs with poor calibration (Spiegelhalter *P* < .001). Retraining the algorithm improved AUROC to 0.92 (95% CI, 0.91-0.93) and AUPRC to 0.66 (95% CI, 0.63-0.68). Number needed to screen in the top risk tiers was 366 for the external model and 200 for the retrained model; the lower number indicates better performance. Domain validation showed AUROC of 0.90 (95% CI, 0.90-0.91) and AUPRC of 0.01 (95% CI, 0.01-0.01), and temporal validation showed AUROC of 0.75 (95% CI, 0.72-0.78) and AUPRC of 0.003 (95% CI, 0.003-0.005).

**Conclusions and Relevance:**

In this cohort study of active-duty Navy service members, a civilian suicide attempt risk model was externally validated. Retraining and updating with DoD-specific variables improved performance. Domain and temporal validation results were similar to external validation, suggesting that implementing an external model in US Navy primary care clinics may bypass the need for costly internal development and expedite the automation of suicide prevention in these clinics.

## Key Points

**Question:**

How well does a civilian-based suicide risk model generalize in US Navy primary care?

**Findings:**

In this cohort study with 260 583 service members, domain and temporal validation showed internal retraining and external validation had similar performance. Updating with US Navy–specific factors added minimal improvement.

**Meaning:**

These findings suggest that civilian-based risk models might generalize to military health settings; prior to transferring risk models, external validation might demonstrate adequate performance in new settings and avoid costly internal development.

## Teaser

This cohort study externally validates and updates a statistical suicide risk model initially developed in a civilian setting with an emphasis on primary care for use among US Navy service members.

## Introduction

Suicide remains a costly and complex challenge for the US military.^1^ Military veterans in the United States are significantly more at risk of attempting suicide than the general population.^2^ While active-duty service members have historically had comparable suicide rates with the general population, suicide rates for service members have increased at a faster rate than the general population since 2015.^3^ The largest population of service members are young and enlisted (ie, early in their careers) and have higher rates of deaths from suicide that account for the majority of attempts.^3,4,5^ In addition, these suicide attempts are more likely to result in death.^6,7,8^ Even more challenging, disclosure of suicidal ideation by service members may have career-altering consequences, increasing the likelihood of covert distress.^9,10,11,12^

To better understand factors that might increase suicide risk in the military, numerous studies have analyzed protective and risk factors for service members.^13,14,15,16^ All service members have been shown to have lower risk when they are focused on a clear mission such as during deployment, yet they experience a higher risk during the postdeployment adjustment period, and this risk increase continues into retirement.^17,18,19^

US service members have contact with primary care practitioners in the month before suicide more than half the time.^20,21,22^ Primary care practitioners seek tools to aid in identifying at-risk patients whom they otherwise may miss.^23^ While those who die from suicide are more likely to perceive treatment barriers, they are also more likely to use health care.^24,25^ Thus, significant attention has been paid to predicting suicide using health care data to guide preventive decisions both within and outside of the military, as meta-analyses suggest traditional screening tools might be inaccurate.^26,27,28,29^ Within the military, the Army Study to Assess Risk and Resilience in Servicemembers (STARRS) has published the best known corpus of such models.^30,31,32^ Broadly, the biomedical literature includes examples of models to predict the emergence, presence, and imminence of suicides.^33,34,35^

To implement a suicide risk model, multidisciplinary teams must develop, validate (test), replicate, and then use models in clinical settings to ensure robustness and continued accuracy.^36,37,38^ In parallel, teams must design effective decision support using risk model data to improve prevention.^39,40,41^ The effort to accomplish these steps is significant. Prior to novel model development, testing externally validated models might result in clinically feasible performance with less time and expense.^42^ These models might also be retrained, such that their input predictors are reweighted to reflect the new setting, or updated, such that novel predictors available only in the new setting are added to the initial models.^43^ Notably, models trained in different settings, such as civilian health systems, might generalize to perform well in military health systems.

In this study, we externally validated a civilian-based statistical risk model currently prompting suicide preventive clinical decision support at Vanderbilt University Medical Center (VUMC) using data from multiple primary care settings serving US Navy sailors.^44,45,46^ We then compared model performance after retraining the model on US Navy primary care data and subsequently updated the model with novel factors only relevant in military health systems that were informed by Department of Defense (DoD)–specific health care variables and demographics, including expanded race and ethnicity categories, present only in DoD sources. The transferability of risk models remains an important research question generally and has clinical implications in suicide risk prediction specifically. We sought to understand how transferring a model trained on a heterogeneous civilian cohort to military settings affects its ability to project suicide attempt risk.

## Methods

This retrospective cohort study included encounters for US Navy active-duty service members who received care at Naval Medical Center Portsmouth (NMCP), Naval Branch Health Clinics (NBHCs), and TRICARE Prime Clinics (TPCs) between 2007 through 2017. Demographic characteristics for this population during the study period were obtained from the Defense Manpower Data Center (DMDC). NMCP NBHCs and TPCs provide routine care for service members and their families. The VUMC, NMCP, and Florida State University institutional review boards approved this study. Informed consent was waived given the study’s minimal risk and its reliance on deidentified records and data collected in routine care. This study adhered to the Strengthening the Reporting of Observational Studies in Epidemiology (STROBE) guidelines.^47^

### Overview

In this cohort study of active-duty Navy service members, we externally validated, retrained, and updated random forest models of suicide attempt risk. In addition, we performed domain and temporal analyses with random forests. An overview of these methods and relevant acronyms can be found in Table 1.

**Table 1.** Key to Model Experiments and Commonly Used Terms

Key	Description
NMCP	NMCP provides emergency, outpatient, and inpatient care and is the parent of the NBHCs and TPCs in this study
NBHCs	Outpatient care clinics used for routine care
TPCs	Outpatient care clinics for acute care injuries
External model	Previously published random forest model trained at VUMC and tested on NMCP visits with all available prior Navy health record data for each visit^44^
Retrained model	Random forest model trained identically as external model using all visits to NBHCs and TPCs (with all prior Navy record data for those visits) and validated with cross validation
Calibrated model	Retrained model with a subsequent layer of calibration using logistic calibration
Updated model	Either the retrained or calibrated model trained again with additional Navy specific features
Domain validation	Random forest trained identically to the external model only at NBHC Little Creek, validated with bootstrapping, and tested at other NBHCs
Temporal validation	Random forest trained identically to the external model with 2 y of NBHC and TPC visits and tested on following 3 y

Abbreviations: NBHC, Naval Branch Health Clinics; NMCP, Naval Medical Center Portsmouth; TPC, TRICARE Prime Clinic; VUMC, Vanderbilt University Medical Center.

### Model and Data Sources

The cohort of Naval health system data was preprocessed based on feature engineering from the published, validated civilian-based model.^44^ Data were obtained from the Navy and Marine Corps Public Health Center, EpiData Center. One data set was created for all NMCP visits and then split in appropriate subsets. In prior work, the civilian-based predictive model was trained at VUMC using the Harrell bias correction method using a case-control data set comprised of electronic health records (EHRs). Case status was determined by 2 suicide experts who manually identified documentation of suicide attempts from 5543 candidate medical records and joining this with a heterogeneous mix of patients seen at VUMC for any reason contemporaneously.^44,48^ Model predictors included counts of diagnostic *International Statistical Classification of Diseases, Tenth Revision, Clinical Modification *(*ICD-9-CM*) codes filtered through Center for Medicare & Medicaid Services (CMS) Hierarchical Condition Categories; counts of Anatomical Therapeutic Chemical level 4 medications; demographic characteristics; and counts of emergency, inpatient, and outpatient visits for each of the 5 years prior to prediction (eTable 2 in Supplement 1).^49,50,51^ This process was repeated in this study with military health system data.

Military health system data were taken from the Standard Inpatient Data Record, the Comprehensive Ambulatory/Professional Encounter Record, the TRICARE Encounter Data Non-Institutional records, and the Theater Medical Data Store. These data sources comprehensively recorded Navy-wide inpatient encounters, ambulatory records, out-of-network care, and medical care received while in the military theater of operation (eg, Operation Iraqi Freedom and Operation Enduring Freedom). *ICD-10-CM* codes, used as input features for the model, were transposed into *International Classification of Diseases, Ninth Revision, Clinical Modification *(*ICD-9-CM*) codes using the *ICD-9-CM* to *ICD-10-CM* General Equivalence mapping from CMS.^52^ Medications were sourced from the Pharmacy Detail Transaction Service, where all pharmacy transactions are logged.

The study data set was prepared recordwise, in which each encounter (record) for each patient (participant) was right-censored at the time of the encounter to replicate clinical data that would have been available to a predictive model running at that time. Demographic variables were retrieved from the most recent record before the prediction date; otherwise the prediction date was discarded. Body mass index (BMI) variables were omitted because of low availability. Race and ethnicity were limited to African American or Black; Asian American; Hispanic; non-Hispanic; White; and unknown, as used in the civilian-based model. All other race and ethnicity variables were encoded as other (eTable 1 in Supplement 2).

### Outcomes and Model Evaluation

The outcomes of interest were *ICD-9-CM* and *ICD-10-CM* suicidal behavior codes recorded on the day of the visit or during the next 30 days. Suicidal behavior codes were defined by the National Center for Health Statistics.^53^ All *ICD-9-CM* suicidal behavior codes used were identical to those used during the development of the statistical model at VUMC.

### Statistical Analysis

To evaluate model performance, discrimination was assessed using area under the receiver operating curve (AUROC), area under the precision recall curve (AUPRC), and Brier score. Calibration was assessed using Spiegelhalter *z*-test statistic, with a significance level of .05. Values less than this significance level indicate miscalibration. Risk concentration was performed and numbers needed to screen (NNS) were calculated for each quantile of risk. The Spiegelhalter *z *test was performed using R package rms version 6.3-0 (R Project for Statistical Computing).

#### Retraining and Updating

After external validation of the civilian-based model on Naval health system data, the random forest model was retrained using 5-fold cross-validation using all visits to NBHCs and TPCs. Retraining was nested record-wise, in which patients were split into training, calibration, and testing sets and all records for each patient were sampled together to avoid training and testing on records for the same patients. The initial training set in each fold was cut from 80% to 75% to create a 5% independent calibration set. Recalibration was performed using logistic calibration.

Model updating added US Navy demographics and DoD extender *ICD-9-CM* and unique *ICD-10-CM* codes as features (eTable 2 in Supplement 1).^54^ Armed Forces Qualification Test (AFQT) categories, time in service, rank group, marital status, education level, and expanded race and ethnicity categories were used as recorded by DMDC (eTable 1 in Supplement 2). Sex and age were the only demographic features present in the external model that were not added or expanded in the retrained model. Broadly, race and ethnicity variables were included having found to be important in the external model.

#### Feature Importance

We compared feature importance weights across external (civilian), retrained, and updated models. Impurity, a common measure of importance used here, measured the amount of variance in the responses at the terminal nodes of the random forest when including or excluding a given feature. Higher variance suggested higher importance. We note impurity assesses importance but not direction, ie, an important feature might be associated with higher or lower risk of the target in question.

#### Temporal and Domain Validation

To test temporal and domain effects, a second domain model was retrained using all visits to NHBC Little Creek (NBHC-LC). This domain validation strategy was intended to simulate a common scenario of training on a given site’s data with external validation at different sites in a larger health system. The NBHC-LC model was tested at the remaining NBHCs and TPCs and on all NBHC and TPC visits that did not belong to a person who visited NBHC-LC during the study period. Harrell bias correction, modified to test on held out bootstrap samples, similar to Efron 0.632 and 632+ methods, was used to assess performance at NBHC-LC.^55^ A third temporal model was retrained using 2 years of NBHC and TPC visits and tested with the next 3 years of visits on both the same service members, and different service members, than those the model was trained on previously.

## Results

Demographic characteristics of service members were determined at the beginning of receiving care at both NMCP and NMCP NBHCs and TPCs (Table 2). Of the 260 583 service members, 206 412 (79.2%) were male; 104 835 (40.2%) were aged 20 to 24 years; and 9458 (3.6%) were Asian, 56 715 (21.8%) were Black or African American, and 158 277 (60.7%) were White; 200 568 (77.0%) visited an NMCP NBHC or TPC. In total, the Navy health system data set contained 6 476 555 visits to NMCP after 442 789 visits (6.4%) were removed for lack of demographic characteristics. Of NMCP visits, 255 089 (3.9%) were emergency visits, 233 497 (3.6%) were inpatient visits, and 5 987 969 (92.5%) were outpatient visits. Visits to NMCP NBHCs and TPCs accounted for 2 418 393 (37.3%) of all NMCP visits and were all outpatient. Of NMCP NBHC and TPC visits, 19 694 (0.8%) were to TPCs.

**Table 2.** Demographics of US Navy Active-Duty Service Members at NMCP and NMCP NBHCs and TPCs Included in Study Data, 2007 to 2017

Characteristic	Service members, No. (%)	
	NMCP (n = 260 583)	NBHCs and TPCs (n = 200 568)
Age, y		
17-19	26 509 (10.17)	17 867 (8.91)
20-24	104 835 (40.23)	76 535 (38.16)
25-29	57 342 (22.01)	46 947 (23.41)
30-34	31 158 (11.96)	25 052 (12.49)
35-39	23 254 (8.92)	19 069 (9.51)
40-44	11 546 (4.43)	9974 (4.97)
≥45	5939 (2.28)	5124 (2.55)
Sex		
Female	54 171 (20.79)	44 306 (22.09)
Male	206 412 (79.21)	156 262 (77.91)
Race		
American Indian or Alaska Native, Black or African American	2009 (0.77)	1548 (0.77)
American Indian or Alaska Native, White	8440 (3.24)	6464 (3.22)
American Indian or Alaskan Native	9428 (3.62)	7295 (3.64)
Asian	9458 (3.63)	6914 (3.45)
Black or African American	56 715 (21.76)	44 033 (21.95)
Native Hawaiian or Other Pacific Islander	2157 (0.83)	1600 (0.8)
Unknown	7281 (2.79)	5525 (2.75)
White	158 277 (60.74)	122 080 (60.87)
Other^a^	6818 (2.62)	5109 (2.56)
Ethnicity		
Filipino	4263 (1.64)	3070 (1.53)
Latin American with Hispanic descent	9266 (3.56)	6950 (3.47)
Mexican	5025 (1.93)	3724 (1.86)
Unknown	25 237 (9.68)	18 788 (9.37)
No ethnicity	172 558 (66.22)	134 337 (66.98)
Other Hispanic descent	17 896 (6.87)	13 683 (6.82)
Puerto Rican	2915 (1.12)	2333 (1.16)
US or Canadian Indian Tribes	5713 (2.19)	4338 (2.16)
Other^a^	17 710 (6.82)	13 345 (6.65)
Marital status		
Married	118 547 (45.49)	96 708 (48.22)
Never married	141 636 (54.35)	103 497 (51.6)
Other^a^	400 (0.15)	363 (0.18)
Education level		
Adult education diploma	2459 (0.94)	1884 (0.94)
Associate’s degree	8940 (3.43)	7335 (3.66)
Baccalaureate degree	24 431 (9.38)	18 935 (9.44)
Completed 1 semester of college, no high school diploma	5048 (1.94)	3928 (1.96)
Doctorate degree	1838 (0.71)	1210 (0.6)
High school diploma	192 126 (73.73)	147 338 (73.46)
Master’s degree	6189 (2.38)	5121 (2.55)
Non–high school graduate	1860 (0.71)	1456 (0.73)
Test-based equivalency diploma	4432 (1.7)	3513 (1.75)
Unknown	11 737 (4.5)	8669 (4.32)
Other^a^	1523 (0.58)	1179 (0.59)
AFQT category		
I	14 495 (5.56)	10 901 (5.44)
II	89 880 (34.49)	70 790 (35.29)
III A	61 575 (23.63)	46 764 (23.32)
III B	55 126 (21.15)	42 058 (20.97)
IV	1386 (0.53)	1152 (0.57)
Unknown or not applicable	38 121 (14.63)	28 903 (14.41)
Rank		
E1-E3	10 720 (4.11)	8386 (4.18)
E4-E6	101 712 (39.03)	70 761 (35.28)
E7-E9	107 980 (41.44)	89 507 (44.63)
O1-O3	15 582 (5.98)	13 682 (6.82)
W1-W3	23 805 (9.14)	17 565 (8.76)
O4, W4, or higher	784 (0.3)	667 (0.33)
Time in service, y		
<1	39 591 (15.19)	26 869 (13.4)
1-2	27 193 (10.44)	21 880 (10.91)
3-4	21 661 (8.31)	17 646 (8.8)
5-9	11 725 (4.5)	10 631 (5.3)
10-14	49 559 (19.02)	38 335 (19.11)
15-19	49 802 (19.11)	43 304 (21.59)
20-24	57 320 (22.0)	38 470 (19.18)
>24	3732 (1.43)	3433 (1.71)

Abbreviations: AFQT, Armed Forces Qualification Test; E, enlisted; NBHC, Naval Branch Health Clinics; NMCP, Naval Medical Center Portsmouth; O, officer; TPC, TRICARE Prime Clinic; W, warrant officer.

^a^ Other categories are listed in eTable 1 in Supplement 2.

At the beginning of receiving care at NMCP NBHCs and TPCs, service members were slightly older, more frequently married, and had more military experience than those receiving care at NMCP (Table 2). No apparent race, ethnicity, or education differences were found between participants visiting NMCP and NMCP NBHCs and TPCs. Of the 260 583 service members to visit NMCP during this time period, 6529 (2.5%) had a recorded suicidal behavior, of whom 2653 (40.6%) had a recorded suicidal behavior on the day of or within 30 days of a visit. Of the 6 476 555 visits to NMCP, 16 715 (0.26%) preceded a suicidal behavior (Table 3). After the data set was developed, and the external model applied to it, performance was assessed.

**Table 3.** Performance of the Civilian-Based External Suicide Risk Model at NMCP and NBHCs and TPCs

Location	Service members, No.	Service member cases, No. (%)	Visits, No.	Visit cases, No. (%)	AUROC (95% CI)	AUPRC (95% CI)	Brier score (95% CI)	Spiegelhalter *z*-test statistic (95% CI)	Spiegelhalter *z*-test *P* value (95% CI)
NMCP	260 583	2653 (1.02)	6 476 555	16 715 (0.26)	0.73 (0.72 to 0.74)	0.013 (0.010 to 0.014)	0.10 (0.10 to 0.10)	−1018.7 (−1028.4 to −1008.8)	<.001 (<.001 to <.001)
NMCP clinics together									
NBHCs	200 299	647 (0.32)	2 398 699	1400 (0.06)	0.77 (0.74 to 0.79)	0.004 (0.003 to 0.005)	0.08 (0.08 to 0.08)	−680.2 (−684.5 to −677.9)	<.001 (<.001 to <.001)
TPCs	4610	30 (0.65)	19 694	56 (0.28)	0.86 (0.78 to 0.90)	0.024 (−0.003 to 0.034)	0.18 (0.17 to 0.18)	−33.7 (−36.3 to −29.4)	<.001 (<.001 to <.001)
Individual NBHCs									
NBHC Naval Station Sewells	128 405	375 (0.29)	1 172 985	737 (0.06)	0.75 (0.72 to 0.77)	0.003 (0.001 to 0.003)	0.08 (0.08 to 0.08)	−475.2 (−477.9 to −474.0)	<.001 (<.001 to <.001)
NBHC Oceana	54 247	123 (0.23)	481 859	257 (0.05)	0.75 (0.71 to 0.80)	0.006 (0.000 to 0.009)	0.07 (0.07 to 0.07)	−305.2 (−306.9 to −302.6)	<.001 (<.001 to <.001)
NBHC Little Creek	61 007	111 (0.18)	444 927	230 (0.05)	0.82 (0.79 to 0.88)	0.005 (0.000 to 0.008)	0.08 (0.08 to 0.08)	−292.4 (−294.6 to −290.0)	<.001 (<.001 to <.001)
NBHC Dam Neck	29 061	50 (0.17)	175 514	102 (0.06)	0.76 (0.73 to 0.83)	0.016 (−0.035 to 0.029)	0.07 (0.06 to 0.07)	−189.6 (−191.8 to −187.6)	<.001 (<.001 to <.001)
NBHC Naval Shipyard Norfolk	19 399	20 (0.10)	50 531	24 (0.05)	0.93 (0.90 to 0.99)	0.014 (−0.057 to 0.024)	0.07 (0.07 to 0.07)	−96.8 (−98.8 to −95.3)	<.001 (<.001 to <.001)
NBHC Yorktown	5610	12 (0.21)	46 662	22 (0.05)	0.73 (0.50 to 0.96)	0.022 (−0.100 to 0.042)	0.07 (0.07 to 0.08)	−95.8 (−99.0 to −93.6)	<0.001 (<0.001 to <0.001)
NBHC Chesapeake	3292	20 (0.61)	26 221	28 (0.11)	0.85 (0.79 to 0.99)	0.007 (0.001 to 0.011)	0.12 (0.11 to 0.12)	−61.5 (−66.0 to −59.4)	<0.001 (<0.001 to <0.001)

Abbreviations: AUPRC, area under the precision recall curve; AUROC, area under the receiver operating characteristic curve; NBHC, Naval Branch Health Clinics; NMCP, Naval Medical Center Portsmouth; TPC, TRICARE Prime Clinic.

NMCP and TPCs had a greater service member and visit prevalence than NBHCs. Visit case prevalence was consistent across the NBHCs. Discrimination in terms of AUPRC was consistent across sizable NBHCs and increased at smaller NBHCs and TPCs. NMCP, NBHCs, and TPCs all had uncalibrated performance from the external algorithm. For reference, performance of the external civilian-based model when tested enterprise-wide had an AUROC of 0.836, Brier score of 0.009, and poor initial calibration, as reported previously.^45^

### Model Retraining, Calibration, and Updating

For NMCP NBHCs and TPCs, 5-fold cross validation was performed with and without DoD- and Navy-specific variables (Table 4). These variables were race (24 categories), ethnicity (23 categories), marital status, education level, AFQT categories, service rank, and time in service. DoD-specific *ICD* variables added included *ICD-9-CM* extenders related to traumatic brain injury (TBI) and *ICD-10-CM* codes tracking the severity, presence, and history of TBI. TBI is known to affect suicide risk.^56^ A full added feature list is available within eTable 2 in Supplement 1.

**Table 4.** Mean Performance of the Retrained, Updated, and Calibrated Suicide Risk Models at Naval Branch Health Clinics and TRICARE Prime Clinics

Metric	Retrained	Updated	Calibrated and retrained	Calibrated and updated
AUROC	0.92 (0.91 to 0.93)	0.92 (0.91 to 0.93)	0.92 (0.91 to 0.93)	0.920 (0.91 to 0.93)
AUPRC	0.62 (0.60 to 0.65)	0.66 (0.63 to 0.68)	0.62 (0.60 to 0.65)	0.66 (0.63 to 0.68)
Brier	3.74 × 10^−4 ^(3.53 × 10^−4^ to 3.96 × 10^−4^)	3.52 × 10^−4^ (3.35 × 10^−4^ to 3.73 × 10^−4^)	3.18 × 10^−4^ (2.96 × 10^−4^ to 3.38 × 10^−4^)	2.93 × 10^−4^ (2.71 × 10^−4^ to 3.14 × 10^−4^)
Spiegelhalter *z*-test statistic	−9.50	−10.41	0.84	0.76
Spiegelhalter *z*-test *P* value	<.001	<.001	.41	.45

Abbreviations: AUPRC, area under the precision recall curve; AUROC, area under the receiver operating characteristic curve.

Mean AUROC and AUPRC within NMCP clinics were 0.92 (95% CI, 0.91-0.93) and 0.62 (95% CI, 0.60-0.65) across the 5 folds before the addition of DoD- and Navy-specific variables. After the addition of DoD- and Navy-specific variables, discrimination improved marginally with a mean AUROC of 0.92 (95% CI, 0.91-0.93) and a mean AUPRC of 0.66 (95% CI, 0.63-0.68).

Logistic calibration was applied to each fold. Mean Spiegelhalter *z*-test statistic *P* value and Brier score before calibration were <.001 and 0.0004, respectively (Table 4). Calibration was successful, as indicated by an improved Spiegelhalter z-test statistic *P* value and Brier score of .41 and 0.0003, respectively.

NNS in the top decile of risk of the external model at NMCP NBHCs was 366, compared with 200 for the updated model (eFigure in Supplement 2). The civilian-based model had a lower NNS than the updated model in the bottom decile, although it contained fewer cases. NNS in each decile refers to the numbers of individuals who would need to receive a test to identify 1 individual who will have the outcome in question.

### Feature Importance

The external, retrained, and updated model features were ranked by importance (impurity) and compared (Table 5). Both the external and retrained models had 1631 features, while the updated model had 1949 (eTable 2 in Supplement 1). Important external model features included age, past suicidal behavior, recent visits, mood disorders, psychotropics, and substance abuse. Simplified race, from the external model, was not important for the retrained or updated model discrimination, while simplified ethnicity, DoD rank, and DoD marital status were important.

**Table 5.** Comparison of the Model Features Between the External, Retrained, and Updated Algorithms

Description	Rank^a^		
	External	Retrained	Updated
Important to external			
Age	1	11	11
Suicide and self-inflicted poisoning by tranquilizers and other psychotropic agents	2	99	116
Suicide and self-inflicted poisoning by other specified drugs and medicinal substances	3	50	68
VUMC race	4	1248	1386
VUMC ethnicity	5	29	44
Outpatient visits (previous year)	6	1	1
Inpatient visits (previous year)	7	4	4
Major depressive affective disorder, recurrent episode, severe, without mention of psychotic behavior	8	56	72
Suicide and self-inflicted poisoning by analgesics, antipyretics, and antirheumatics	9	148	163
Emergency visits (previous year)	10	8	7
Diazepines, oxazepines, thiazepines, and oxepines	11	38	52
Other and unspecified alcohol dependence, unspecified	12	25	39
Major depressive affective disorder, single episode, unspecified	13	22	26
Posttraumatic stress disorder	14	44	57
Important to updated			
Outpatient visits (previous year)	6	1	1
Outpatient visits (1-2 y ago)	20	2	2
Outpatient visits (2-3 y ago)	45	3	3
Inpatient visits (previous year)	7	4	4
Propionic acid derivatives	32	5	5
Anilides	27	6	6
Emergency visits (previous year)	10	8	7
Outpatient visits (3-4 y ago)	72	7	8
Opium alkaloids and derivatives	74	9	9
Selective serotonin reuptake inhibitors	25	10	10
Age	1	11	11
DoD time in service	NA^b^	NA^b^	12
Expectorants	140	12	13
Other specified counseling	NA^b^	NA^b^	14
DoD race	NA^b^	NA^b^	15

Abbreviations: DoD, Department of Defense; NA, not applicable; VUMC, Vanderbilt University Medical Center.

^a^ Lower number for rank indicates higher importance.

^b^ Feature was added in the updated model.

Other DoD- and Navy-specific variables, including medical examinations, counseling, time in service, expanded race, expanded ethnicity, and AFQT categories, were important for updated model discrimination. Outpatient visits, pain relievers, antidepressants, hormones, and antibiotics were also important. Posttraumatic stress disorder (PTSD), alcohol dependence, and benzodiazepines were consistently important between the external and updated models. Updating the algorithm after retraining did not affect the most important features compared with retraining alone.

### Temporal and Domain Validation

Discrimination for domain validation at NBHC-LC, obtained from bootstrapping, was much greater than both the external model and the locations outside of NBHC-LC with an AUROC of 0.96 (95% CI, 0.91-1.00) and AUPRC of 0.58 (95% CI, 0.39-0.77) (eTable 3 in Supplement 2). Discrimination was greatest at NBHC Oceana and at the TPCs. Discrimination dropped to an AUROC of 0.90 (95% CI, 0.90-0.91) and an AUPRC of 0.01 (95% CI, 0.01-0.01) at non–NBHC-LC locations when service members who visited NHBC-LC were removed. Calibration was poorest at NBHCs Oceana and Yorktown.

Discrimination for temporal validation resembled external validation when the third model was trained with early NBHC and TPC visits and then tested with later visits. Performance for service members seen by the third model had the same discrimination (AUROC, 0.74 [95% CI, 0.71-0.77]; AUPRC, 0.003 [95% CI, 0.003-0.005]) compared with not seen (AUROC, 0.75 [95% CI, 0.72-0.78]; AUPRC, 0.003 [95% CI, 0.003-0.005]).

## Discussion

This cohort study was the first to our knowledge to comprehensively validate, retrain, and update models of suicide risk in US Navy health system data. Primary findings include an assessment of the performance of a civilian-based suicide risk model and a comparison of retrained and updated models specific for a US Navy population. External validation of predictive algorithms has been relatively rare.^57^ We note a generally low rate of suicidal behaviors (2.5%) in this cohort across the study period, a positive finding for military health.

Consistent discrimination with poor calibration was obtained from applying the external model to NMCP, NBHCs, and TPCs. Retraining with US Naval data resulted in improved apparent performance with the key caveat that internal validation (retraining) had higher performance (more optimism) than external validation, but external validation was a more rigorous test. Both internal and external validation were performed in this study. Logistic calibration was successfully able to recalibrate model performance as expected. Model updating was associated with marginal improvement compared with only retraining and indicates a need for further DoD-specific feature collection and engineering. For example, mental health assessments were present in a small percentage of these overall study data, but they might prove important in specific clinical settings or in cohorts enriched for their use.

US Navy data, however, were still important for model performance. Expanded race was helpful, while the externally defined simplified race was not helpful. Valid risk factors were seen in the most important model features. Notable differences between the external and updated model features were conditions, such as PTSD, which when reported during active duty, may result in an earlier transition.^58^ PTSD and other known risk factors, including Vitamin A derivatives, were within the top 1% and 2% of features.^59,60^ Caution should be taken when interpreting importance values, and we underline our reported statistics remain correlative, not causal, in inference.

Greater performance than in external validation was found in the domain validation, with optimistic apparent performance at the training location, NBHC-LC. However, when service members who visited NBHC-LC were removed, performance dropped to a level only slightly better than external validation. This removal ensured performance increases did not result from model bias toward service members available to the model in retraining. In the temporal validation, both service members seen by the model and not seen by the model had the same discrimination. Both were equal to external validation, indicating that adopting an external model in some settings may have similar prospective performance to retraining with less time and cost.

Implications of this study suggest a civilian-based suicide risk model alone would achieve feasible NNS for an automated risk assessment first-pass to be supported or refuted by clinician evaluation and judgment.^45^ In clinical use, a model like this one might have application to prompt further screening or discussion of suicide to prevent covert distress. It also might serve to guide populational health management by quantifying burden of suicidality by clinical setting, geography, or other relevant grouping. With respect to modeling implications, retraining improved precision of this model further (lowering NNS and false positives) while model updating did not, in this case, improve performance notably. Of note, some highly relevant assessments (eg, the mental health assessment) were not present in sufficient quantities in this cohort to justify updating at scale. Future work to study cohorts with these and other assessments is indicated.

US Navy data in this study are comprehensive with respect to ascertainment of health care data, including purchase claims outside NMCP, pharmacy data, and study outcomes. These data included transitions through clinics and hospitals, into the military theater, and into intensive therapy or treatment regimens. These strengths contrast with most civilian health systems in which care is open and health care information exchange is generally lacking, with key exceptions being payer-based systems and states in which significant investment in information exchange has been made.^61^ This study used multiple validation strategies, including replicating the mode in predictive modeling, in which a model is trained in one location and validated elsewhere, either across differing clinical settings (domain validation), time, or geography.^62^

### Limitations

This study has limitations. Different external model performance between VUMC and the US Navy, suggested by AUROC, contrasted with the markedly optimistic performance metrics obtained from retraining, may indicate that a retrained model may be more overfit. Markers of treatment and utilization, or normally undertaken clinical scenarios, may drive model performance instead of imminent risk factors. Self-reported data obtained during routine assessments and known risk factors for suicide in the military, which would likely improve the model, such as predeployment nightmares, were not explicitly represented.^63^ Further exploration of available DoD-specific features including more specific, localized model development, eg, within specific clinical settings, might demonstrate more value to model updating with US Navy data. Data from within assessments (eg, Pre/Post Deployment Health Assessments) were not used as features, only that information that the assessments were performed.

Further refinement of the retraining approach and application of the model to unseen data are needed. In this study, the retrained algorithm, using a smaller fraction of the data focused on primary care clinics, was able to generalize to other clinics similar to the external model. Work toward assessing the feasibility of obtaining the comprehensive data used here at run time in the US Navy is needed. Additionally, deep learning algorithmic approaches might facilitate transfer learning in which a pretrained model (civilian-based EHR) is improved through learning on the novel data set (US Naval EHR).

## Conclusions

In this cohort study of active-duty Navy service members, we externally validated, retrained, and updated civilian-based statistical models of suicide attempt risk. The external civilian-based risk model estimated risk of suicide attempt on the day of or within 30 days of every visit to NMCP, NBHCs, and TPCs. The model had similar discrimination to models trained and updated in temporal and domain validation, suggesting retraining might not be necessary prior to next steps in deployment of algorithms like these in military clinical settings. Evaluating prior published models prior to time-consuming novel model development might facilitate similar applied modeling efforts.

## Notes

Supplement 1. **eTable 2.** External, Retrained, and Updated Model Features

Supplement 2. **eTable 1.** All Department of Defense (DoD) Categories for Race, Ethnicity, Marital Status, and Education Level **eFigure.** Case Prevalence and Numbers Needed to Screen (NNS) for the External and Updated Models **eTable 3.** Performance of the NBHC-LC Suicide Risk Model for Domain Validation at Other NBHCs and TPCs and at NBHC-LC via Bootstrapping

Supplement 3.
Data Sharing Statement
