# Supplementary material for: External Validation and Updating of a Statistical Civilian-Based Suicide Risk Model in US Naval Primary Care
Source: JAMA Netw Open. 2023 Nov 8;6(11):e2342750. doi: 10.1001/jamanetworkopen.2023.42750 (PMC10632956; doi:10.1001/jamanetworkopen.2023.42750)
Supplement: Supplement 2. — eTable 1. All Department of Defense (DoD) Categories for Race, Ethnicity, Marital Status, and Education Level eFigure. Case Prevalence and Numbers Needed to Screen (NNS) for the External and Updated Models eTable 3. Performance of the NBHC-LC Suicide Risk Model for Domain Validation at Other NBHCs and TPCs and at NBHC-LC via Bootstrapping [file jamanetwopen-e2342750-s002.pdf]

Supplemental Online Content

Ripperger MA, Kolli J, Wilimitis D, et al. External validation and updating of a statistical civilian-based suicide risk model in US Naval primary care. *JAMA Netw Open*. 2023;6(11):e2342750. doi:10.1001/jamanetworkopen.2023.42750

eTable 1. All Department of Defense (DoD) Categories for Race, Ethnicity, Marital Status, and Education Level

eFigure. Case Prevalence and Numbers Needed to Screen (NNS) for the External and Updated Models

eTable 3. Performance of the NBHC-LC Suicide Risk Model for Domain Validation at Other NBHCs and TPCs and at NBHC-LC via Bootstrapping

This supplemental material has been provided by the authors to give readers additional information about their work.

© 2023 Ripperger MA et al. *JAMA Network Open.*

eTable 1. All Department of Defense (DoD) Categories for Race, Ethnicity, Marital Status, and Education Level

Categories labeled as "Other" for the VUMC external model and for main text Table 2 demographics are shown.

Demographic	All Department of Defense (DoD) Categories	VUMC External Model "Other"	Table 2 Demographics "Other"
Race	American Indian/Alaska Native, Asian	American Indian/Alaska Native, Asian	American Indian/Alaska Native, Asian
Race	American Indian/Alaska Native, Asian, Black or African Ameri	American Indian/Alaska Native, Asian, Black or African Ameri	American Indian/Alaska Native, Asian, Black or African Ameri
Race	American Indian/Alaska Native, Asian, Native Hawaiian or oth	American Indian/Alaska Native, Asian, Native Hawaiian or oth	American Indian/Alaska Native, Asian, Native Hawaiian or oth
Race	American Indian/Alaska Native, Asian, White	American Indian/Alaska Native, Asian, White	American Indian/Alaska Native, Asian, White
Race	American Indian/Alaska Native, Black or African American	American Indian/Alaska Native, Black or African American	NA
Race	American Indian/Alaska Native, Black or African American, Na	American Indian/Alaska Native, Black or African American, Na	American Indian/Alaska Native, Black or African American, Na
Race	American Indian/Alaska Native, Black or African American, Wh	American Indian/Alaska Native, Black or African American, Wh	American Indian/Alaska Native, Black or African American, Wh
Race	American Indian/Alaska Native, Native Hawaiian or other Paci	American Indian/Alaska Native, Native Hawaiian or other Paci	American Indian/Alaska Native, Native Hawaiian or other Paci
Race	American Indian/Alaska Native, White	American Indian/Alaska Native, White	NA
Race	American Indian/Alaskan Native	American Indian/Alaskan Native	NA
Race	Asian	NA	NA
Race	Asian, Black or African American	Asian, Black or African American	Asian, Black or African American
Race	Asian, Black or African American, Native Hawaiian or other P	Asian, Black or African American, Native Hawaiian or other P	Asian, Black or African American, Native Hawaiian or other P
Race	Asian, Black or African American, White	Asian, Black or African American, White	Asian, Black or African American, White
Race	Asian, Native Hawaiian or other Pacific Islander	Asian, Native Hawaiian or other Pacific Islander	Asian, Native Hawaiian or other Pacific Islander
Race	Asian, Native Hawaiian or other Pacific Islander, White	Asian, Native Hawaiian or other Pacific Islander, White	Asian, Native Hawaiian or other Pacific Islander, White
Race	Asian, White	Asian, White	Asian, White
Race	Black or African American	NA	NA
Race	Black or African American, Native Hawaiian or other Pacific	Black or African American, Native Hawaiian or other Pacific	Black or African American, Native Hawaiian or other Pacific
Race	Black or African American, White	Black or African American, White	Black or African American, White

© 2023 Ripperger MA et al. *JAMA Network Open.*

Demographic	All Department of Defense (DoD) Categories	VUMC External Model "Other"	Table 2 Demographics "Other"
Race	Native Hawaiian or other Pacific Islander	Native Hawaiian or other Pacific Islander	NA
Race	Native Hawaiian or other Pacific Islander, White	Native Hawaiian or other Pacific Islander, White	Native Hawaiian or other Pacific Islander, White
Race	Unknown	Unknown	NA
Race	White	NA	NA
Ethnicity	Aleut	Aleut	Aleut
Ethnicity	Asian Indian	Asian Indian	Asian Indian
Ethnicity	Chinese	Chinese	Chinese
Ethnicity	Cuban	NA	Cuban
Ethnicity	Eskimo	Eskimo	Eskimo
Ethnicity	Filipino	Filipino	NA
Ethnicity	Guamanian	NA	Guamanian
Ethnicity	Japanese	Japanese	Japanese
Ethnicity	Korean	Korean	Korean
Ethnicity	Latin American with Hispanic Descent	NA	NA
Ethnicity	Melanesian	Melanesian	Melanesian
Ethnicity	Mexican	NA	NA
Ethnicity	Micronesian	Micronesian	Micronesian
Ethnicity	Unknown	NA	NA
Ethnicity	No Ethnicity	No Ethnicity	NA
Ethnicity	Other (Undefined)	Other (Undefined)	NA
Ethnicity	Other Asian Descent	Other Asian Descent	Other Asian Descent
Ethnicity	Other Hispanic Descent	NA	NA
Ethnicity	Other Pacific Island Descent	Other Pacific Island Descent	Other Pacific Island Descent
Ethnicity	Polynesian	Polynesian	Polynesian
Ethnicity	Puerto Rican	NA	NA
Ethnicity	US or Canadian Indian Tribes	US or Canadian Indian Tribes	NA
Ethnicity	Vietnamese	Vietnamese	Vietnamese
Marital Status	Annulled	NA	Annulled
Marital Status	Divorced	NA	Divorced

© 2023 Ripperger MA et al. *JAMA Network Open.*

Demographic	All Department of Defense (DoD) Categories	VUMC External Model "Other"	Table 2 Demographics "Other"
Marital Status	Married	NA	NA
Marital Status	Unknown	NA	Unknown
Marital Status	Never Married	NA	NA
Marital Status	Widowed	NA	Widowed
Education Level	Adult education diploma	NA	NA
Education Level	Associate degree	NA	NA
Education Level	Baccalaureate degree	NA	NA
Education Level	Completed one semester of college, no high school diploma	NA	NA
Education Level	Correspondence school diploma	NA	Correspondence school diploma
Education Level	Doctorate degree	NA	NA
Education Level	First professional degree	NA	First professional degree
Education Level	High school certificate of attendance	NA	High school certificate of attendance
Education Level	High school diploma	NA	NA
Education Level	Home study diploma	NA	Home study diploma
Education Level	Master's degree	NA	NA
Education Level	Non-high school graduate	NA	NA
Education Level	Occupational program certificate	NA	Occupational program certificate
Education Level	Post master's degree	NA	Post master's degree
Education Level	Professional nursing diploma	NA	Professional nursing diploma
Education Level	Secondary school credential near completion	NA	Secondary school credential near completion
Education Level	Test-based equivalency diploma	NA	NA
Education Level	Unknown	NA	NA

© 2023 Ripperger MA et al. *JAMA Network Open.*

eFigure 1. Case Prevalence and Numbers Needed to Screen (NNS) for the External and Updated Models

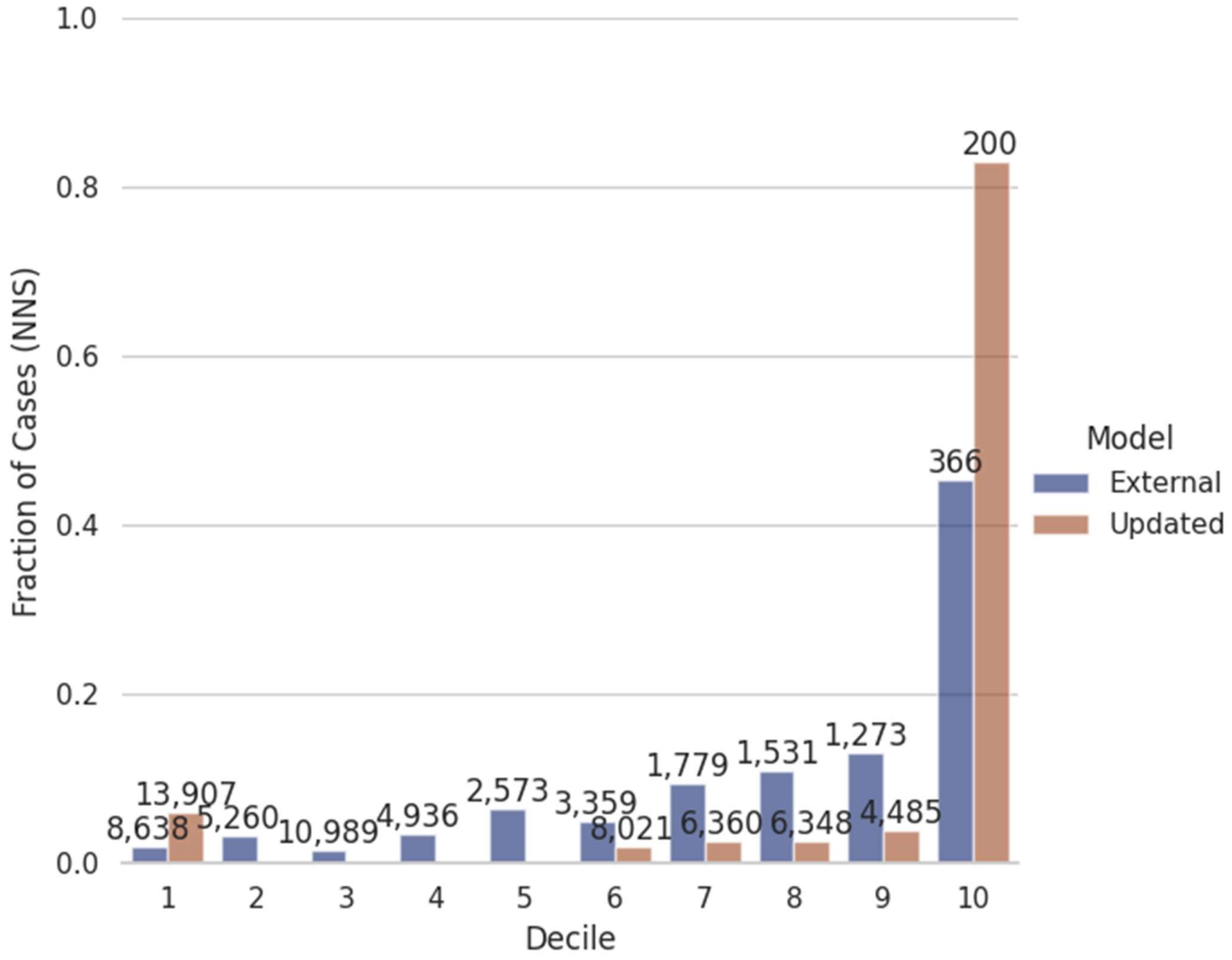

© 2023 Ripperger MA et al. *JAMA Network Open.*

eTable 3. Performance of the NBHC-LC Suicide Risk Model for Domain Validation at Other NBHCs and TPCs and at NBHC-LC via Bootstrapping

Location	AUROC	AUPRC	Brier	Spiegelhalter's z-Test Statistic	Spiegelhalter's z-Test p-Value
NBHC-LC	0.96	0.58	0.00061	-6.84	0.006
**Non-NBHC-LC Records**					
Non-NBHC-LC NBHCs / TPCs	0.90	0.070	0.0010	-2.47	0.01
Non-NBHC-LC NBHCs / TPCs w/o NBHC-LC Persons	0.90	0.011	0.0011	1.13	0.26
**Non-NBHC-LC Locations**					
NBHC Chesapeake	0.95	0.053	0.0019	-1.23	0.22
NBHC Dam Neck	0.86	0.045	0.00092	-0.743	0.48
NBHC Naval Station Sewells	0.90	0.059	0.0011	-0.353	0.72
NBHC Naval Shipyard Norfolk	0.91	0.0078	0.00073	-0.598	0.55
NBHC Oceana	0.89	0.12	0.00082	-3.61	<0.001
NBHC Yorktown	0.91	0.070	0.00062	-3.77	<0.001
TRICARE Outpatient Chesapeake	0.99	0.92	0.00062	-1.62	0.11
TRICARE Outpatient Virginia Beach	0.86	0.17	0.0058	3.61	<0.001

© 2023 Ripperger MA et al. *JAMA Network Open.*
