# Supplementary material for: External Validation and Updating of a Statistical Civilian-Based Suicide Risk Model in US Naval Primary Care
Source: JAMA Netw Open. 2023 Nov 8;6(11):e2342750. doi: 10.1001/jamanetworkopen.2023.42750 (PMC10632956; doi:10.1001/jamanetworkopen.2023.42750)
Supplement: Supplement 3. — Data Sharing Statement [file jamanetwopen-e2342750-s003.pdf]

Data Sharing Statement

Data
Data available: No

Additional Information
Explanation for why data not available: Data are sensitive, military health system derived health records
